# Therapeutic potential of N-acetylcysteine as an antiplatelet agent in patients with type-2 diabetes

**DOI:** 10.1186/1475-2840-10-43

**Published:** 2011-05-21

**Authors:** Kyle R Gibson, Tim J Winterburn, Fiona Barrett, Sushma Sharma, Sandra M MacRury, Ian L Megson

**Affiliations:** 1 Free Radical Research Facility, Department of Diabetes & Cardiovascular Science, University of the Highlands & Islands, Inverness, Scotland, UK; 2 Highland Clinical Research Facility, University of the Highlands & Islands, Centre for Health Science, Inverness, Scotland, IV2 3JH, UK

**Keywords:** type 2 diabetes, platelets, thrombosis, antioxidants, glutathione, N-acetylcysteine

## Abstract

**Background:**

Platelet hyperaggregability is a pro-thrombotic feature of type-2 diabetes, associated with low levels of the antioxidant glutathione (GSH). Clinical delivery of *N*-acetylcysteine (NAC), a biosynthetic precursor of GSH, may help redress a GSH shortfall in platelets, thereby reducing thrombotic risk in type-2 diabetes patients. We investigated the effect of NAC *in vitro*, at concentrations attainable with tolerable oral dosing, on platelet GSH concentrations and aggregation propensity in blood from patients with type-2 diabetes.

**Methods:**

Blood samples (*n *= 13) were incubated (2 h, 37°C) with NAC (10-100 micromolar) *in vitro*. Platelet aggregation in response to thrombin and ADP (whole blood aggregometry) was assessed, together with platelet GSH concentration (reduced and oxidized), antioxidant status, reactive oxygen species (ROS) generation, and plasma NOx (a surrogate measure of platelet-derived nitric oxide; NO).

**Results:**

At therapeutically relevant concentrations (10-100 micromolar), NAC increased intraplatelet GSH levels, enhanced the antioxidant effects of platelets, and reduced ROS generation in blood from type-2 diabetes patients. Critically, NAC inhibited thrombin- and ADP-induced platelet aggregation *in vitro*. Plasma NOx was enhanced by 30 micromolar NAC.

**Conclusions:**

Our results suggest that NAC reduces thrombotic propensity in type-2 diabetes patients by increasing platelet antioxidant status as a result of elevated GSH synthesis, thereby lowering platelet-derived ROS. This may increase bioavailability of protective NO in a narrow therapeutic range. Therefore, NAC might represent an alternative or additional therapy to aspirin that could reduce thrombotic risk in type-2 diabetes.

## Background

Oxidative stress is implicated in the aetiology and complications of type-2 diabetes [1]. Elevated reactive oxygen species (ROS) production is a feature of platelets in type-2 diabetes [2] and contributes to hyperaggregability associated with the disease [3,4] through both depression of intra-platelet antioxidant status and reduced synthesis and bioavailability of anti-thrombotic nitric oxide (NO) [5]. Oxidative stress also results in endothelial dysfunction associated with atherosclerosis and subsequent thrombotic complications [6], compounded in type-2 diabetes by a sustained hypercoagulable state and alterations in the fibrinolytic pathway. Since the revelation that aspirin fails to show benefit in primary prevention of cardiovascular events in diabetes [7,8], there is renewed urgency in finding alternative antithrombotic therapies.

Glutathione (GSH) is an abundant, key endogenous antioxidant that is depressed in platelets from patients with type-2 diabetes, contributing to hyperaggregability [9]. Intramuscular GSH administration increases levels of protective NO in platelets from type-2 diabetes patients, with a concomitant decrease in plasma levels of the fibrinolytic inhibitor, plasminogen activator inhibitor-1 [10]. However, GSH is a poor candidate for oral therapy on account of peptide digestion in the intestinal tract and poor membrane penetration. *N*-acetylcysteine (NAC), on the other hand, is a well-recognised therapy to redress acute GSH depletion in acetaminophen overdose. Therapeutic roles for NAC have also been proposed in a range of clinical conditions, although there are some doubts about its efficacy [11].

In the diabetes arena, oral NAC has been shown to improve endothelial function in a rat model of diabetes [12] and to reduce endothelial activation and oxidative stress markers [13] and blood pressure [14] in patients with type-2 diabetes. Very high concentrations (3 mmol/l) augment NO-mediated inhibition of platelet aggregation in blood from obese patients *in vitro *[15]. Our own study *in vitro *indicated that much lower concentrations of NAC (10-100 μmol/l) depress platelet function in blood from healthy volunteers, an effect that was associated with increased intra-platelet GSH and inhibition of oxidative stress [16].

Here, we tested the hypothesis that NAC, at plasma concentrations achievable through oral dosing, inhibits platelet hyperaggregability in blood from patients with type-2 diabetes, and set out to examine the biochemical background to any beneficial effects observed.

## Methods

### Clinical Study Design and Population

Eligible subjects with type-2 diabetes were recruited through local General Practitioners. At an initial screening visit, venous blood (9 ml) was collected from the antecubital fossa into lithium heparin-containing tubes (Sarstedt Ltd, Leicester, UK) to screen lipid and renal status (Piccolo Lipid and Renal Panels, respectively; Abaxis, Darmstadt, Germany), whilst a finger-prick sample was obtained for HbA_1C _determination (DCA 2000+ Analyser; Bayer, Newbury, UK).

Subjects that met the inclusion criteria for the study (Table 1) returned to the Highland Clinical Research Facility within two weeks of the screening visit. Venous blood (250 ml) was drawn from the antecubital fossa using an intravenous cannula (BD Venflon 18G, BD Medical, Oxford, UK) and transferred into tubes containing 3.8% trisodium citrate or 7.5 g/l K_3_EDTA. Blood cell counts (Beckman Coulter ACT10; Beckman Coulter, High Wycombe, UK) were conducted on the trisodium citrate sample, in triplicate, primarily in order to assess platelet number in the samples used for aggregometry and biochemical assays.

**Table 1:** Patient characteristics

*Parameter*	
Age (years)	59.2 ± 3.7
Gender (M:F)	10:5
BMI (kg/m^2^)	32.2 ± 1.9
HbA_1C _(%)	6.5 ± 0.3
Total Cholesterol (mmol/l)	4.4 ± 0.2
LDL Cholesterol (mmol/l)	2.5 ± 0.1
HDL Cholesterol (mmol/l)*	1.4 ± 0.1
Triglycerides (mmol/l)	1.3 ± 0.1
Creatinine (μmol/l)	91.5 ± 6.5
Fasting glucose (mmol/l)	7.9 ± 0.5
Haemoglobin (g/l)	117 ± 5.0
Haematocrit (%)	37.1 ± 1.4
Platelets (× 10^9 ^cells/l)	208.1 ± 16.1

Abbrevations: *, calculated; BMI, body mass index; F, female; HbA_1C_, glycosylated haemoglobin; HDL, high-density lipoprotein; LDL, low-density lipoprotein; M, male. Data are expressed as mean ± sem (*n *= 15).

On study days, following a 12 h fast, volunteers arrived at the Highland Clinical Research Facility (between 9-10 am to minimise diurnal variation of antioxidant levels and blood constituents, particularly platelets). All subjects provided informed written consent prior to participation. The study was approved by the Highland Research Ethics Committee and was conducted in accordance with the Declaration of Helsinki and its amendments.

### Statistical Power

A review of the literature pertaining to the various assays conducted in this project identified platelet aggregometry (thrombin agonist) as the least powerful technique. For a significance level of 5% and a power of 80% for detecting a change in aggregation of 30%, 15 subjects were recruited. Complete data sets were achieved for n = 13 (aggregometry) and n = 10-12 for biochemical measures.

### Blood Preparation

Citrated blood samples were divided into four groups: a control with no NAC, and three treatment groups (10, 30 and 100 μmol/l NAC). Blood samples were incubated with NAC (or vehicle control) for 2 h at 37°C.

### Washed platelet preparation

Platelet rich plasma (PRP) was first prepared from citrated whole blood by centrifugation (200 g, 10 min, room temperature). Platelets were washed by centrifuging (1200 *g*, 10 min, room temperature) 2 ml PRP aliquots treated with prostacyclin (PGI_2_; 30 ng/ml). The plasma was subsequently aspirated and replaced with an equal volume of citrated (3.8% trisodium citrate) Tyrode buffer (composition in mmol/l: 137 NaCl, 2.7 KCl, 1.05 MgSO_4_, 0.4 NaH_2_PO_4_, 12.5 NaHCO_3_, 5.6 Glucose, 10 HEPES and 0.8 CaCl_2_; pH 7.4) containing 30 ng/ml PGI_2_, prior to gentle resuspension of the platelets with the aid if a micropestle. Following a second centrifugation and aspiration of the supernatant, the platelets were gently resuspended in Tyrode buffer in the absence of PGI_2_. Platelet preparations were standardized to 100 × 10^9 ^platelets/l using citrated Tyrode buffer for use in all experiments requiring washed platelets.

### Platelet Aggregation

Platelet aggregation was assessed in a 4-channel impedance aggregometer (Chrono-Log Model 700 Lumi-Aggregometer; LabMedics, Manchester, United Kingdom). Diluted blood (500 μl whole blood + 500 μl 0.9% saline) was pre-warmed at 37°C for 5 min. Aggregation was initiated by addition of ADP (Labmedics, Manchester, UK; 0.625 - 10 μmol/l) or thrombin (0.125 - 1 U/ml) and progress tracked as the area subtended by the resultant increase in impedance over 6 min. Experiments were conducted at 37°C with a stirring speed of 1000 rpm and impedance gain of 0.05. No specific anticoagulant was added to the thrombin-treated preparations in order to preserve the full pharmacological impact of thrombin in these experiments; the aggregatory response to thrombin is strictly a combination of platelet and coagulation cascade activation.

### Glutathione (GSH) Determination

Total GSH (i.e. GSH+GSSG) and GSSG levels were measured in platelet extracts using the GSH reductase enzyme method; the protocol is described in fine detail in a previous publication [17]. This assay involves the thiol-mediated conversion of 5,5'-dithio-bis (2 nitrobenzoic acid) (DTNB; Ellman's reagent) to 5-thio-2-nitrobenzoic acid (TNB), detectable at λ = 412 nm. The test is specific to GSH on the basis of the specificity of the GSH reductase enzyme to GSH: the rate of accumulation of TNB is proportional to the concentration of GSH in the sample. Briefly, platelets from untreated and NAC-treated (2 h, 37°C) blood samples (collected in EDTA) were isolated by centrifugation (200 *g; *10 min) to obtain PRP, prior to further centrifugation steps (2 × > 1200 *g*, 4°C, 10 min) to first isolate and then wash the platelet pellet. Supernatant was then removed and the platelet pellet exposed to metaphosphoric acid (5%; 4°C). After centrifugation, the cell lysate was collected and diluted 1:1 with KPE buffer prior to addition of freshly prepared DTNB and GSH reductase solutions, prepared according to [17]. Following addition of β-NADPH, the absorbance (λ = 412 nm) was measured immediately and at 30 s intervals for 2 min in a 96 well plate. The rate of change in absorbance was compared to that for GSH standards. The method for assessment of the GSSG in each sample was identical to that for GSH, but with a prior treatment of the sample with 2-vinylpyridine to react out the reduced GSH at the outset. Protein concentrations in extracts were quantified (Coomassie Protein Assay Kit, Perbio Science UK Ltd, Northumberland, UK).

### Platelet EPR Spectrometry

EPR is a specific technique to enable the measurement of free radicals: only entities with unpaired electrons are detected by the technique. The highly reactive nature of oxygen-centred free radicals (e.g. superoxide and hydroxyl) means that they do not exist sufficiently long in samples to be measured directly. Instead, high concentrations of a suitable 'spin-trap' are used to compete with other potential reactants for free radicals: the product of the reaction of a radical with such a trap forms a stable radical (spin-adduct) that is detectable by EPR spectrometry. The intensity of the signal increases as the spin-adduct accumulates at a rate that is proportional to the rate of free radical production.

Potential antioxidant effects of washed platelets were assessed using an *in vitro *model of oxidative stress. The foundation for the model is that HEPES present in Tyrode buffer is known to spontaneously generate oxygen-centred radicals [18] that are detectable using the cell-permeable spin trap that is specific for oxygen-centred radicals (1-hydroxy-3-carboxy-pyrrolidine, CP-H; Axxora, Nottingham, UK). Briefly, whole blood was incubated (2 h; 37°C) with NAC (10-100 μmol/l) as described above, prior to preparation of washed platelets from the samples (washing removes extracellular NAC and facilitates introduction of radical-generating HEPES in Tyrode buffer). 1 mmol/l CP-H spin-trap was then added to the washed platelets and the EPR signal corresponding to the spin adduct CP^.^, was measured at 30 min intervals for a further 4 h using EPR spectrometry (Miniscope MS200; Magnettech, Germany; parameter settings: B0-field, 3356 G; sweep width, 50 G; sweep time, 30 s; modulation amplitude, 1500 mG; microwave power, 20 mW; microwave frequency, 9.3 GHz; gain, 1). The ability of platelets pre-treated with NAC to effectively compete with the spin trap for oxygen-centred free radicals was assessed by comparison to an untreated control washed platelet preparation and to Tyrode buffer without platelets for each of 12 patient samples.

### ROS Generation: Chemiluminescence

Generation of ROS was determined using lucigenin-derived chemiluminescence in washed platelets. Washed platelets were incubated for 10 min in a Lumi-Aggregometer (Chrono-Log Model 700 Lumi-Aggregometer, LabMedics, Manchester, UK; settings: 37°C; stirring speed 1000 rpm; luminescence gain 2). Lucigenin (125 μmol/l), a luminescent probe for detecting ROS, was incubated with samples for 10 min - the area under the curve for the luminescent signal generated corresponded to the basal level of ROS in each sample. Subsequently, thrombin (1 U/ml) was added to stimulate the ROS release and the chemiluminescent signal quantified over 40 min. Superoxide dismutase (SOD; 300 U/ml) was added to verify the sensitivity of the assay for detecting O_2_^.-^.

### Platelet NO Synthase (NOS)-derived NO metabolites (NO_x_)

Measurement of NO metabolites (NO_x_) in collagen-activated washed platelets was used as a surrogate marker of platelet NOS activity [19]. NO has a half-life of seconds in biological media, making it extremely difficult to measure. The major product of NO oxidation/metabolism is nitrite (NO_2_^-^), which can further oxidise to nitrate in a process that is accelerated by iron centres (e.g. haem). Other potential fates of NO are peroxynitrite (ONOO^-^), which would be expected to isomerise to nitrate or react to form other products (e.g. nitrotyrosine) and S-nitrosothiols. The assay used in this study does not detect nitrate, but does not discriminate between NO_2_^- ^and S-nitrosothiols (collectively termed NOx from now on). Briefly, samples of treated (NAC; 10-100 μmol/l; 2 h; 37°C) and untreated citrated whole blood were centrifuged, the platelet rich plasma aspirated and washed, as described above. Samples were then divided into two: half was treated with the NOS inhibitor, L-NAME (200 μmol/l; 37°C), for 10 min, whilst the other half remained untreated. Samples were activated with collagen (2 μg/ml; Labmedics, Manchester, UK) and incubated (5 min; 37°C) and snap-frozen. Thawed samples (100 μl) were injected into a purge vessel containing glacial acetic acid and potassium iodide; NO generated in the purge vessel was transferred in a carrier gas (oxygen-free nitrogen) to a chemiluminescence NO Analyser (Sievers 280i NO analyzer; GE Analytical Instruments, Colorado, USA; method of [20]). The difference in NOx detected in the L-NAME-treated and untreated portions for each sample were taken to represent platelet NOS-derived NOx.

### NAC measurement

Total NAC (i.e. oxidized + reduced) was measured in platelet extracts using HPLC with fluorescence detection (Agilent Technologies 1200 Series, South Queensferry, UK) - adapted from [21].

### Statistical Analyses

Results are expressed as mean ± sem. Concentration-response curves were fitted to non-linear regression (sigmoidal dose-response, variable slope), except for calibration curves which were fitted to linear regression. Curve fitting and statistical tests were performed using GraphPad Prism software (version 5.00). Data distribution was assessed using the Kolmogorov-Smirnov test. All data sets followed Gaussian distribution; it was therefore appropriate to use one-way ANOVA to compare more than two groups and one factor. Concentration-response curves were analysed by two-way ANOVA with repeated measures. Dunnett's or Bonferroni *post hoc *tests were conducted, where appropriate, to correct for multiple testing. *P *< 0.05 was considered to be statistically significant.

### Drugs, Chemicals, Reagents and Other Materials

All drugs were obtained from Sigma (Poole, UK) unless otherwise specified. The spin adduct, 4-oxo-tempo, and spin trap, CP-H, were constituted using phosphate-buffered saline; CP-H also contained EDTA (10 mmol/l final concentration).

## Results

### Subject Characteristics

The characteristics of the study population are shown in Table 1.

### Therapeutic Potential of NAC to Reduce Platelet Hyperaggregability

**Figure 1:**
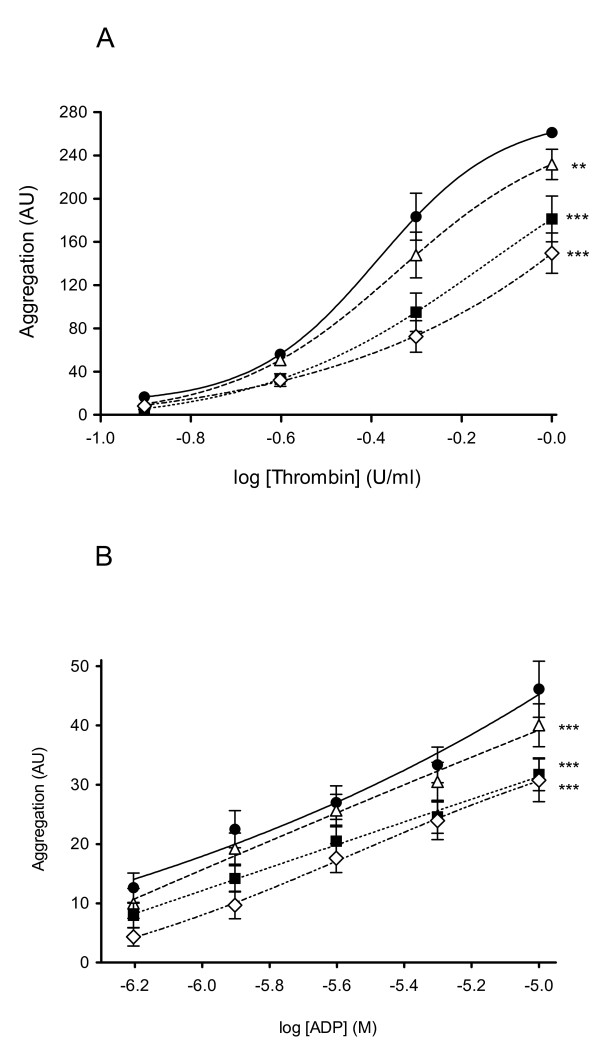
**Therapeutic potential of in vitro additions of NAC to reduce platelet aggregability in whole blood from patients with type-2 diabetes**. Inhibition of (**A**) thrombin- (0.125-1.0 U/ml) and (**B**) ADP (10 μmol/l)-induced platelet aggregation, by NAC (10-100 μmol/l; 2 h, 37°C), (*n *= 13 for both). Black circles = vehicle control; white triangles = 10 μmol/l NAC; black squares = 30 μmol/l NAC; white diamonds = 100 μmol/l NAC. Data are expressed as mean ± sem; statistical analysis was performed by two-way ANOVA with Bonferroni's *post hoc *correction for multiple testing (treatment vs control). **P < 0.01, ***P < 0.001.

Thrombin and ADP both induced aggregation in diluted whole blood preparations, although the maximum aggregation was much greater in thrombin-treated preparations than those exposed to ADP. All three concentrations of NAC (10-100 μ mol/l; 2 h incubation) caused inhibition of thrombin (P < 0.01 for 10 μ mol/l NAC, *P *< 0.001 for 30 and 100 μ mol/l compared to control; two-way ANOVA,) and ADP (*P *< 0.001 for all NAC concentrations compared to control; two-way ANOVA) -induced platelet aggregation in whole blood from patients with type-2 diabetes (Figure 1A and 1B). Blood from all patients (*n *= 13) responded to NAC. The concentration-dependent effects of NAC on inhibiting platelet aggregation were particularly marked for thrombin-induced aggregation (Figure 1A).

### Glutathione Status

**Figure 2:**
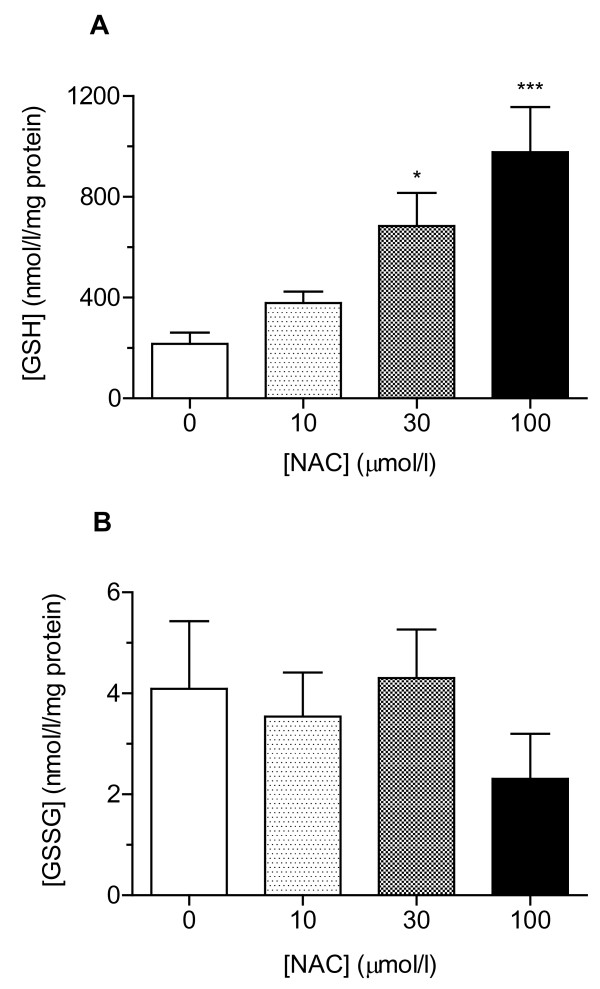
**NAC impact on (**A**) total and (**B**) oxidised glutathione in platelet extracts (*n *= 11)**. Data are expressed as mean ± sem; statistical analysis was performed by one-way ANOVA with Dunnett's *post hoc *test vs control. *P < 0.05; ***P < 0.001.

NAC increased total GSH concentration in a concentration-dependent manner (*P *< 0.0001; Figure 2A), but did not impact on GSSG levels (*P *= 0.52; Figure 2B).

### Antioxidant Effect of Washed Platelets

**Figure 3:**
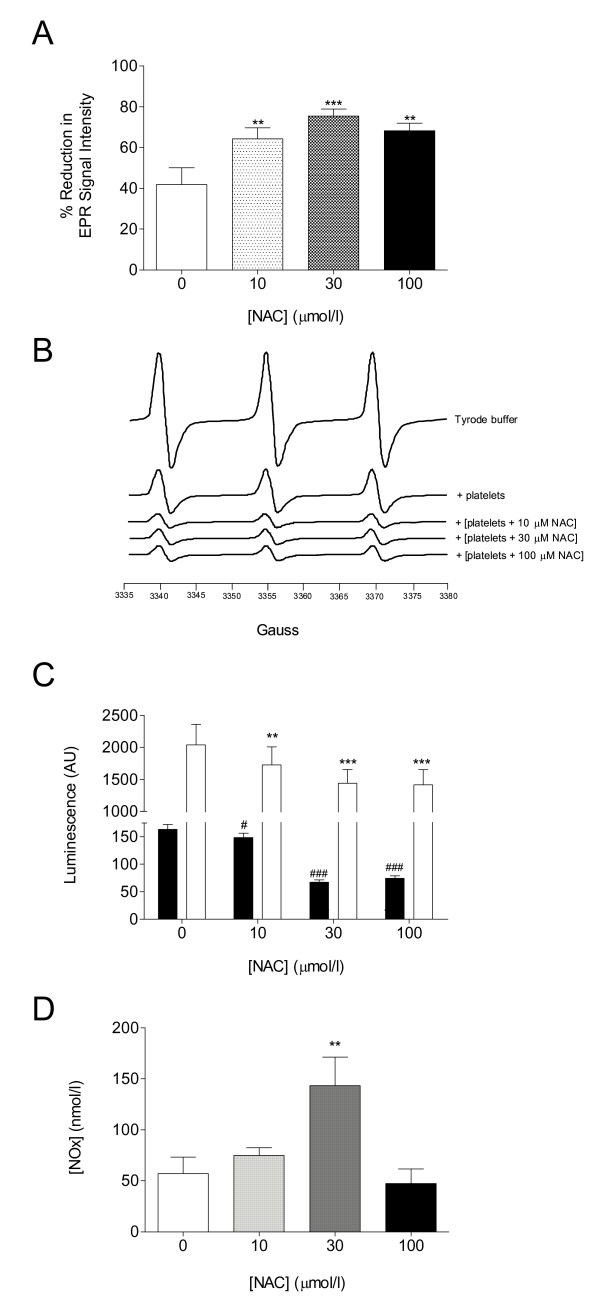
**Effect of in vitro additions of NAC on antioxidant capacity and platelet-derived ROS in platelet samples from patients with type-2 diabetes**. (**A**) Mean data for the effect (*n *= 12); data are expressed as mean ± sem; statistical analysis was performed by one-way ANOVA with Dunnett's *post hoc *test vs control. **P < 0.01; ***P < 0.001. (**B**) Typical 3-line EPR spectra obtained at the 4 h reading for Tyrode's buffer alone and in washed platelets with or without treatment with NAC (10-100 μmol/l; 2 h; 37°C - NAC washed out of supernatant before addition of spin-trap). (**C**) ROS detection in washed platelets. Luminescence detection of ROS in both the basal state (black bars) and following thrombin-stimulation (white bars) of washed platelets (*n *= 12). Data are expressed as mean ± sem; statistical analysis was performed by one-way ANOVA with Dunnett's *post hoc *test vs control for each group (basal and thrombin-activated samples were analysed separately). **P < 0.01; ***P < 0.001 (thrombin-activated vs control): ^#^P < 0.05; ^###^P < 0.001 (basal vs control). **(D) **L-NAME sensitive nitrite detection in collagen activated washed platelets (*n *= 10). Data are expressed as mean ± sem; statistical analysis was performed by one-way ANOVA with Dunnett's *post hoc *test vs control. **P < 0.01.

Washed platelets with no treatment had an antioxidant effect (*P *= 0.01) in most type-2 diabetes patients (*n *= 12). However, washed platelets from a patient subset (*n *= 3) displayed a higher EPR signal intensity than the control (Tyrode buffer), indicating a pro-oxidant activity. Nevertheless, treatment with NAC was increased the antioxidant effect of washed platelets (*P *< 0.0001; Figure 3A) in blood from all patients, most markedly with 30 μ mol/l NAC. Following two cycles of platelet washing, NAC was undetectable in the plasma or in platelet extracts (via fluorescence HPLC, detection threshold ~2 μmol/l). Typical EPR spectra are illustrated in Figure 3B.

### Reactive Oxygen Species Generation

In both non-activated (basal) and thrombin (1 U/ml)-activated platelets (washed preparation), ROS generation was inhibited in a concentration-dependent manner by NAC (*P *< 0.0001; Figure 3C). Activation with thrombin increased ROS generation relative to the corresponding basal levels (*P *< 0.0001). The addition of SOD (300 U/ml; *n *= 6) abolished the luminescence signal (*P *< 0.0001; data not shown).

### Platelet NO Bioavailability

Incubation (2 h, 37°C) with 30 μmol/l NAC increased L-NAME-sensitive NOx levels (×2.6 compared to control; p < 0.05; Figure 3D). The impact of 10 and 100 μmol/l NAC on platelet NOx did not reach statistical significance.

## Discussion

This study shows that NAC, at appropriate plasma concentrations for realistic oral dosing [22], inhibits thrombin- and ADP-induced platelet aggregation in blood from patients with type-2 diabetes. This anti-platelet effect is associated with increased intra-platelet GSH and enhanced antioxidant activity. Moreover, ROS generation from both activated and quiescent platelets decreased with NAC supplementation. Finally, L-NAME-sensitive NOx, a surrogate measure of NO bioavailability, was increased by 30 μmol/l NAC. Overall, our findings suggest that NAC inhibits platelet hyperaggregability in blood from patients with type-2 diabetes by reversing oxidative stress and increasing antioxidant capacity via increased GSH biosynthesis.

The NAC concentrations chosen for this study approximate the peak plasma concentrations achievable with oral doses of 100-1000 mg [22]. The duration of drug exposure *in vitro *was a compromise between the likely peak effect of NAC on intracellular GSH [22] and the maximum period for maintenance of optimal platelet function after sampling (3 h).

In blood from all type-2 diabetes patients studied (*n *= 13), platelet aggregation was inhibited significantly by NAC at all three concentrations, irrespective of whether thrombin (which activates both platelet aggregation and coagulation) or ADP (a specific platelet activator) was the agonist involved. The results are similar in nature (if not mechanism) to those reported for high concentration, short duration exposure to NAC of platelets from obese subjects [15]. In addition, our findings are in accordance with those reported previously in platelets from healthy volunteers [16], although the effect is more dramatic and displays a greater concentration dependence in platelets from the type-2 diabetes patient group compared to controls. Nevertheless, there results suggest a levelling-off of the effect at 100 μmol/l, perhaps suggesting that higher concentrations might not continue to further suppress aggregation.

The suppressed aggregation of platelets treated with NAC was associated with an increase in their capacity to deal with oxidative stress, manifested as lowered ROS production. The fact that SOD abolished the luminescence signal indicates that the majority, if not all, of the ROS detected was ^.^O_2_^-^. This agrees with previous studies highlighting the significance of ^.^O_2_^- ^in altering platelet function [5].

Importantly, the depression in ROS generation is not a direct action of NAC, which we showed previously to be a weak antioxidant in its own right [16]. Indeed, these assays were performed only after two platelet washing cycles to ensure removal of extracellular NAC, and HPLC measures on treated platelet extracts failed to detect intra-platelet NAC (detection threshold ~2 μmol/l). Instead, we surmise that the antioxidant effect of NAC is primarily mediated through its ability, as a biosynthetic precursor, to boost production of intra-platelet GSH, consistent with previous studies in other cell types (for example [23]).

It is perhaps surprising that an acute normalization of the intra-platelet redox-environment should have such rapid effects with respect to reducing platelet aggregability, because much of the oxidative damage caused by ROS (e.g. lipid peroxidation, protein modification) would take much longer to reverse. However, the intra-platelet, constitutive NO synthase (NOS)-mediated synthesis of NO responds rapidly to changes in Ca^2+ ^produced by platelet activation. It has been postulated that release of NO upon platelet activation acts as a negative feedback to further platelet activation/recuitment, thereby raising the threshold of stimulus necessary to cause aggregation, and helping to prevent inappropriate thrombus formation. It follows that loss of NO, through quenching by ROS, could release this physiological brake, thereby encouraging aggregation. Given that the interaction between ROS and NO is very rapid, an increase in the antioxidant capacity within platelets would have an immediate impact on NO bioavailability. Our experiments to measure exclusively NOS-derived NOx in activated platelet preparations indicated that the intermediate concentration of NAC (30 μmol/l) increased NOS-dependent NOx formation by ~2.5-fold. A similar increase in NO bioavailability with NAC was reported in a previous study [14], in which the authors suggested that the effect was due to increased NO synthesis. We make no such assertion, because NO bioavailability is affected by the rates of both NO synthesis (via NOS) and inactivation, particularly by oxygen-centred free radicals like ^.^O_2_^-^. Interestingly, in our experiments, the effect was only seen at 30 μmol/l NAC; it was not matched in samples treated with either 10 or 100 μmol/l NAC, perhaps suggesting a very narrow therapeutic window for this action. In light of these results, we speculate that continuing to increase NAC in an effort to improve efficacy might prove counter-productive.

Whilst the GSH-dependent antioxidant hypothesis might go some way to explain the inhibitory effect of NAC in this study, the lack of consistent concentration-dependence between the various measures (ROS generation, platelet antioxidant activity, GSH levels, NOS-dependent NOx) suggests that the antioxidant activity, which is apparently saturated by ~30 μmol/l, is supplemented by alternative mechanisms of action at higher concentrations. One possible explanation is that NAC may be having direct effects at the receptor level in platelets, as seen in similar studies using human macrophages and vascular smooth muscle cells [24,25].

## Conclusions

NAC, at pharmacologically-relevant concentrations, significantly inhibits platelet hyperaggregability in type-2 diabetes patients *in vitro*. The likely mechanism is through enhancement of platelet GSH levels, suppression of ROS and, possibly, increased protection of the anti-aggregation local messenger, NO. This study displays the clinical potential of NAC in treating platelet hyperaggregability in type-2 diabetes. Moreover, in view of both the ineffectiveness of aspirin in this patient group [7,8], and recent findings relating to a protective role for NAC in hypertension and endothelial function [13,14], a clinical trial of oral NAC in a type-2 diabetes context is justified.

## Abbreviations

ADP: adenosine diphosphate; ANOVA: analysis of variance; BMI: body mass index; CPH: 1-hydroxy-3-carboxy-pyrrolidine; DTNB: 5,5'-dithio-bis (2 nitrobenzoic acid); EPR: electron paramagnetic resonance; GSH: glutathione; GSSG: oxidsied glutathione; HPLC: high performance liquid chromatography; LDL: low density lipoprotein; L-NAME: N^ω^-nitro-L-arginine methyl ester; NAC: N-acetylcysteine; NO: nitric oxide; NOS: NO synthase; NOx: [nitrite + S-nitrosothiols]; PRP: platelet rich plasma; PGI_2_: prostacyclin (prostaglandin I_2_); TNB: 5-thio nitrobenzoic acid.

## Competing interests

The authors declare that they have no competing interests.

## Authors' contributions

KG - researched data, contributed to discussion, wrote manuscript. TW - researched data, contributed to discussion, reviewed/edited manuscript. FB - researched data, recruited patients. SS - researched data. SM - contributed to discussion, reviewed manuscript. IM - contributed to discussion, wrote manuscript, reviewed/edited manuscript. All authors read and approved the final version.
